# Retinal vascular abnormalities and their associations with cardiovascular and cerebrovascular diseases: a Study in rural southwestern Harbin, China

**DOI:** 10.1186/s12886-020-01407-y

**Published:** 2020-04-06

**Authors:** Junwei Wang, Fei Leng, Zhijian Li, Xianling Tang, Hua Qian, Xiaoguang Li, Yi Zhang, Xuedong Chen, Haitao Du, Ping Liu

**Affiliations:** 1 Eye Hospital, First Affiliated Hospital, Harbin Medical University, Harbin, 150001 China; 2 Department of Pharmacology, College of Pharmacy, Harbin Medical University, Harbin, China

**Keywords:** Retinal vascular abnormalities, Risk factors, Cross-sectional study, Prevalence, Rural population, Cardiovascular and cerebrovascular diseases

## Abstract

**Background:**

Limited data is available on retinal vessel morphology in the north China. The study aimed to evaluate the prevalence of retinal vascular abnormalities (RVAs) and investigate their associations with the self-reported diagnosis of cardiovascular and cerebrovascsular diseases (CCVds) in a rural adult population of northeast China.

**Methods:**

A population-based, cross-sectional study was conducted, using the cluster random sampling method. One eye of each participant was photographed with a non-mydriatic fundus camera. RVAs including focal and general arteriolar narrowing (FAN and GAN), arteriovenous nicking (AVN), arteriolar sheathing (AS), and retinopathy were evaluated. Data on self-reported diagnosis of cardiovascular and cerebrovascular diseases and status of smoking and alcohol drinking were obtained from questionnaires.

**Results:**

Among the 6267 participants with an age ≥ 50 years, photographs were obtained of 99.2%, with quality sufficient to perform retinal evaluations in 82.5%. The prevalence of FAN, AVN, AS, retinopathy and GAN were 9.1, 8.9, 5.0, 6.6 and 6.2%, respectively. All the retinal lesions were associated with hypertension (all *P* < 0.01). After adjusting for age, gender, and left/right eyes, hypertension, hyperlipidaemia, diabetes mellitus, habits of past or current smoking and alcohol consumption, AVN was strongly associated with the self-reported diagnosis histories of coronary heart diseases(CHD) (OR, 1.44; 95% CI, 1.09, 1.89) and retinopathy was significantly associated with a self-reported diagnosis of stroke (OR, 2.05; 95% CI, 1.18, 3.57).

**Conclusions:**

The overall prevalence of retinal microvascular abnormalities in this population was relatively higher than that reported in other regions of the world. Retinopathy is associated with the self-reported diagnosis of stroke while AVN was associated with the self-reported diagnosis of CHD, but the remaining retinal lesions were not consistently associated with CCVds. Thus, an examination of retinal microvascular characteristics may offer clues to CCVds and could be a potentially novel biomarkers for CCVds risk.

## Background

Retinal vascular abnormalities (RVAs, a general term for retinal lesions such as focal and generalized retinal arteriolar narrowing (FAN and GAN), arteriovenous nicking (AVN), arteriolar sheathing (AS), and retinopathy) [1–4], have increasingly considered when assessing the risk of cardiovascular and cerebrovascular diseases (CCVds) [4]. Previous population-based studies have reported a strong link between RVAs and clinical stroke [5–11], and there is also evidence that RVAs are predictive of clinical coronary artery disease events [12, 13]. However, most of the previous studies were conducted in white populations; only one study has included a Chinese population [14]. In contrast, this study found that RVAs are not related to the a self reported of coronary heart disease (CHD) or previous cerebrovascular events, such as stroke.

In the past decades, the development of ocular fundus photography and image-processing software has enabled accurate and reproducible assessment of retinal microcirculatory alterations [3, 15–17]. However, the current range of software programs for measuring retinal vascular changes is not fully automated or easy to use without standardized protocols, training, and additional input by technicians. Thus, these resources are only used as research tools and are not yet widely available for clinical use. Therefore, how to convert retinal vascular imaging from a research tool into a technique that can assess CCVds risk and finally into a clinical tool applicable in daily clinical practice is an urgent problem to be solved.

Considering the role retinal vascular changes may have in the early detection of CCVds and the lack of research on retinal vessel morphology in the adult Asian population, we conducted the present study to assess the RVAs in northeastern China. This study focused on the prevalence of RVAs and their associations with CCVds among subjects aged 50 years or older.

## Methods

### Population and sample ascertainment

**Fig. 1:**
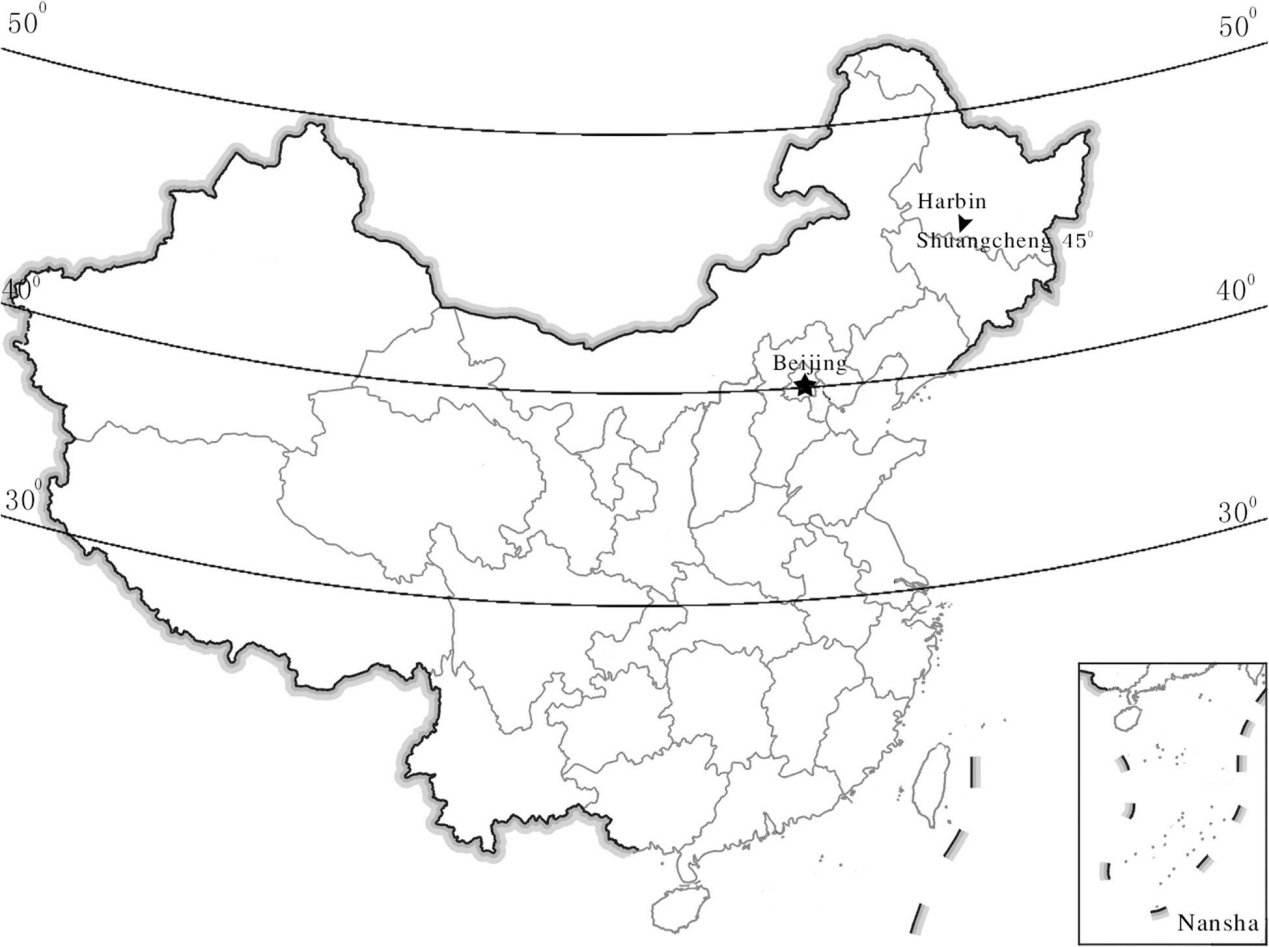
The location of the present survey area, Shuangscheng (the sketch map was generated by Adobe Illustrator CS5)

Shuangcheng (Fig. 1), a specific region of southwestern Harbin, northeast China, that was characterized by cold weather, low elevation, farming communities, and plains, was selected as the survey area. The population of the region is approximately 830,000, with 650,000 people living in rural areas (18 Xiang, 256 villages).

Geographically defined clusters based on village register census data were used as the study sampling frame within each county/district. In this study, a cluster random sampling method, similar to that used in the study by Doumen [18] was employed. The sample size was determined by RVA prevalence (age ≥ 40 years, 0.043, 14] within the allowable error boundary of 20% and a 95% confidence interval (CI) [19], along with assuming an examination response rate of 85% and a design effect of 1.5 to account for inefficiencies associated with the cluster sampling design. In brief, the sample design used village-based clusters of almost the same size (approximately 1000 people). Using streets as dividing lines, villages with populations of more than 1500 people were separated into 2 units, and villages with populations of fewer than 500 people were merged with the closest neighbouring village with populations of fewer than 800 people [20]. Then, 582 sampling units were obtained, from which 35 units were randomly selected. This study included individuals over 50 years old, who comprised approximately 20% of the total population [21]. Finally, 6849 people were eligible for this study.

### Data collection methods

The research protocol was approved by the Medical Ethics Committee of the First Affiliated Hospital of Harbin Medical University (No:201532) and all the subjects provided informed consent, according to the Declaration of Helsinki. Households listings of names of residents ≥50 years of age were obtained from the village registers, followed by door-to-door visits conducted by the enumeration team. Individuals temporarily absent at the time of the household visit were included in the enumeration. Unregistered adults ≥50 years of age were enumerated and included in the study sample if they had been living in the household for at least 6 months. Refusal to provide informed consent was considered as an exclusion criterion.

All participants were administered a questionnaire [22], basic physical examination, and laboratory evaluation, which included basic demographic characteristics (name, age, gender, locality, etc.), medical histories (hypertension, hyperlipemia, diabetes, stroke, cardiovascular and cerebrovascular diseases, smoking status and alcohol consumption), blood pressure measurements, fasting blood glucose measurement and blood lipid measurements. All examinations were carried out in the villages, either in clinics or in the houses of the village committees. Those who did not appear at the examination site were revisited, repeatedly if necessary, by a member of the enumeration team to encourage participation.

### Assessment of hypertension, Hyperlipemia, CCVds and their risk factors

The methods for the assessment of hypertension, CCVds, and their risk factors are highlighted here.self-reported diagnosis histories of CCVds and smoking habits and alcohol consumption were obtained from a questionnaire. Sitting brachial blood pressure was measured three times by trained technicians with a random-zero sphygmomanometer after 5 min of rest. Blood samples were collected between 7:00 and 9:00 a.m. after at least an 8 h overnight fasting. Sterile vacuum tubes with and without ethylenediaminetetraacetic acid were used, and centrifugation was performed within 3-h of blood collection. Serum analysis was performed in the laboratory of the First Affiliated Hospital of Harbin Medical University (quality control of the laboratory was certified and monitored yearly by the Ministry of Health, China). Hypertension was diagnosed, if the systolic blood pressure was ≥140 mmHg or diastolic blood pressure was ≥90 mmHg, or a self-reported diagnosis history of hypertension and antihypertensive therapy was self-reported. Hyperlipemia was diagnosed, if Triglycerides were ≥ 2.0 mmol/L and high-density lipoprotein was ≤1.0 mmol/L, or a self-reported history of hyperlipemia and lipid-lowering therapy was self-reported. Diabetes mellitus was diagnosed, if the fasting glucose was ≥7.0 mmol/ L (≥126 mg/dl) or the use of insulin or oral hypoglycaemic medication was self-reported [22].

### Assessment of retinal vascular abnormalities

**Fig. 2:**
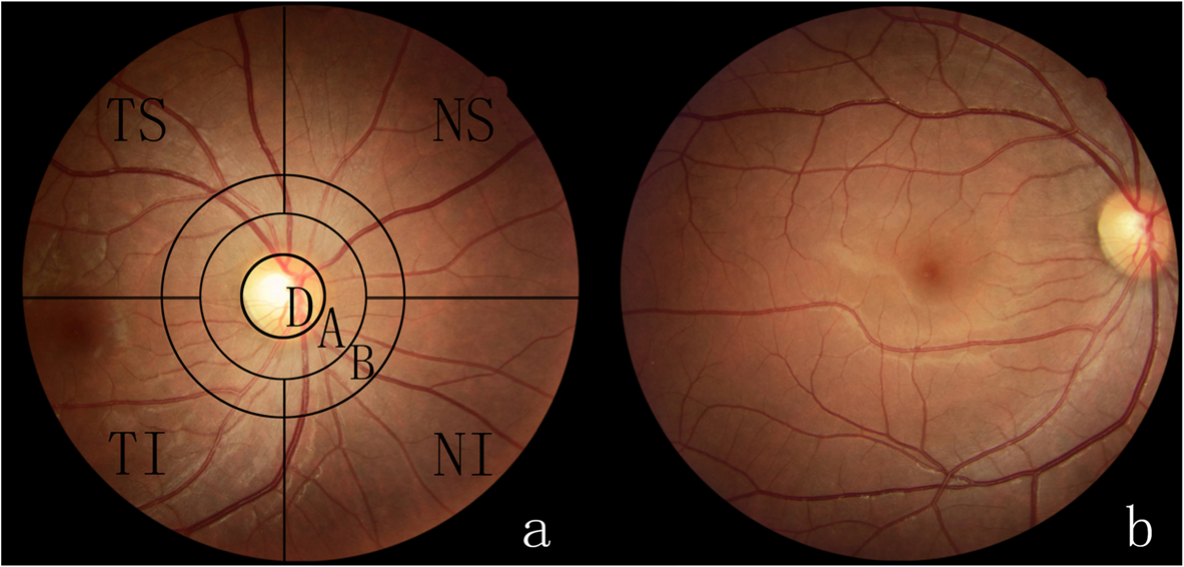
A colour fundus photograph of the right eye of a participant, centred on the optic disc, shows the photographic field definition and the superimposed grid. The grid is composed of three circles concentric with the optic disc: the innermost circumscribes the average disc, the middle one outlines the annulus from the disc margin to ½ disc diameter (DD) from the margin (zone A), and the outer one outlines the annulus from ½ DD to 1 DD from the disc margin (zone B). The four lines radiating from the central circle divide the photograph into four quadrants in relation to the disc: superior temporal (TS), superior nasal (TN), inferior nasal (NI), and inferior temporal (TI). **b**. The colour fundus photograph of the right eye of a participant, centred on the macula

To evaluate RVAs, two 40° non-mydriatic retinal photographs of one eye from each participant were taken using a fundus camera (Type. Classic, 3nethra, Indian). One photograph was centred on the optic disc and another on the fovea centre (Fig. 2 follows the standard Fields 1 and 2 in the Early Treatment of Diabetic Retinopathy Study (ETDRS)) [23]. To achieve balance, if the identification numbers were even, the right eye was chosen; on the contrary, if the identification numbers were uneven, the left eye was chosen. The above retinal photography methods were principally followed the Atherosclerosis Risk in the Community (ARIC) protocol [3].

The inclusion criteria for the study population was as follows: subjects who had clear fundus photographs that could be evaluated were included (including those with previous cataract surgery or previous history of vitrectomy, and intravitreal medications). The exclusion criteria of this study population were as follows: subjects whose fundus photographs were not clear and could not be evaluated; and subjects who lacked relevant data.

The colour photographs (image resolution: 2048*1536 24 bits per pixel, JPG) of each subject were evaluated, according to the evaluation criteria of the ARIC study [3] and ETDRS [23]. The photographs were assessed by two assessors who were trained at the Retinal Vascular Imaging Centre, University of Melbourne (the assessors were blinded to the participant characteristics) using a semi-quantitative manual grading approach for the digital images.

For the evaluation of RVAs, FAN, AVN, AS, retinopathy and GAN were assessed. The grader compared possible abnormalities with standard and example photographs to help determine their presence and severity. The ARIC grid [3] (described in Fig. 2) was applied to divide the retina into standard regions. The regions outside zone A region (zone B and distal regions), FAN, AVN, AS, and GAN in each quadrant were graded. Standard photographs for retinal microvascular signs were selected by a retinal specialist from a standard photographic set developed for the Modified Airlie House Classification of Diabetic Retinopathy [23].

FAN [3] was diagnosed if the artery with a diameter greater than or equal to 50 μm, appeared to narrow, with a diameter is equal to or smaller than 2/3 of its distal and proximal vessel segments. According to the total length of vascular stenosis in the quadrant, less than ½ DD, between ½DD and 2 DD, and 2 DD or more, indicated the different severity levels including mild, moderate and severe, respectively. AVN [3] was diagnosed if both sides of the venous blood column were gradually narrowed at the intersection of the arteries and veins. If the narrowing approached approximately ½ of the blood column, AVN was classified as “mild/moderate.” If the narrowing was equal to or less than ½ of the blood column, AVN was considered as “severe.” AS [3] was considered definite if white sheathing was observed on one side or both sides of retinal vessels. For the assessment of generalized narrowing of the retinal arterioles, the arterial diameter was compared with the corresponding veins, unless the veins were engorged and tortuous. If the arterioles appeared to be narrow in comparison with the veins, GAN was graded as “questionable.” If some arterioles in the eye were markedly narrowed or thready but appeared normal in other quadrants of the fundus, GAN was graded as “definite.” If the arterioles were small and thready throughout the entire eye, GAN was graded as “severe” [14].

For retinopathy, the following lesions were evaluated in each of the four quadrants of the retina: microaneurysms, hemorrhages, soft exudates or cotton wool spots, hard exudates, macular oedema, intraretinal microvascular abnormalities, venous beading, new vessels in the disc or elsewhere, and vitreous haemorrhage. Retinopathy was diagnosed if any of these lesions were definite or probable in any of the four quadrants. The severity of retinopathy was summarized according to the ETDRS severity scale [23].

### Quality control of RVAs assessment

To examine the reproducibility of the assessment of RVAs, 100 pictures were randomly selected and evaluated by two ophthalmologists respectively. Every observer repeated the work 2 weeks later.

### Statistical analysis

The statistical analysis was carried out using SPSS 19.0 (Chicago, IL, USA). The five primary endpoints in the study were FAN, AVN, AS, retinopathy and GAN. We calculated the prevalence of each retinal outcome, according to gender and age group. The association between RVAs (FAN, AVN, AS, retinopathy and GAN) and CCVds and their risk factors was determined using logistic regressions. Odds ratios (ORs) and 95% confidence intervals (CIs) were calculated. All the *P*-values were determined a 2-sided test and were regarded as significant when they were less than 0.05.

## Results

### Participants

A total of 6267 participants received examinations, and the response rate was 91.5%. Fundus photographs were obtained from 99.2% the responding participants, and of photographs (from 5172 participants) were eligible for retinal evaluations. There was no difference in age (*P* = 0.38) and gender (*P* = 0.63) between participants with evaluable photographs (5172) and those with unevaluated photographs (1095) (Table 1). The 5172 participants with evaluable photographs were finally employed for all the following analyses. Of the 5172 participants, 2043 were males and 3129 were females. The average age was 61.25 ± 7.37 years.

**Table 1:** Characteristics of survey population (N,%)

		Total N(%)	Participants with evaluable photographs N(%)	Non-participants or participants with non-evaluable photographs N(%)	*p* value
*Age (Yrs)			61.25 ± 7.37	61.47 ± 7.46	0.38
**Gender	Male	2467 (37.5)	2043 (39.5)	424 (38.7)	0.63
	Female	3800 (62.5)	3129 (60.5)	671 (61.3)	

Footnotes: * Data presented are mean ± standard deviation (SD)

** Chi-square test

### Prevalence of retinal vascular abnormalities

For the entire study population, prevalence of focal arteriolar narrowing (FAN), arteriovenous nicking (AVN), arteriolar sheathing (AS), retinopathy and generalized arteriolar narrowing (GAN), respectively, were 9.1, 8.9, 5.0%,6.6 and 6.2% (Table 2). In general, the prevalence of AS, retinopathy, and GAN increased significantly with age, even after adjusting for gender. After adjusting for age, FAN, AS, and retinopathy were significantly more frequent in males than in females. For all types of retinal vessel abnormalities, no significant differences in the frequencies were found between the left and right eyes(*P* > 0.05, Table 2).

**Table 2:** Prevalence of retinal vascular abnormalities in rural southwestern Harbin,China (%)

		Focal Retinal Vascular Abnormalities				Generalized Narrowing
		Focal arteriolar narrowing	Arteriovenous nicking	Arteriolar sheathing	Retinopathy	
	Total	9.1	8.9	5.0	6.6	6.2
Age (Yrs)†	50–59	8.2	8.2	3.9	5.6	5.7
	60–69	9.7	8.8	5.4	6.5	5.7
	70–79	9.7	12.3	6.6	10.4	9.1
	80–91	13.3	6.7	10.7	13.3	10.7
*P* value		0.626	0.096	0.034	< 0.001	0.036
Gender‡	Male	11.3	9.9	6.8	6.3	7.1
	Female	7.7	8.3	3.8	6.9	5.6
*P* value		< 0.001	0.183	< 0.001	0.017	0.161
Eye	Left	8.7	9.6	5.2	7.2	6.5
	Right	9.5	8.3	4.7	6.1	5.9
P value		0.339	0.117	0.370	0.122	0.829

Footnotes: Test of trend, comparing % between groups

†Adjusted for gender,comparing adjusted % age groups. ‡Adjusted for age, comparing adjusted % between male and female

### Association of RVAs with CCVds and their risk factors

Logistic regression models were constructed to determine the association between each retinal outcome and CCVds and their risk factors. All types of RVAs were more frequent in persons with hypertension than in those without (Table 3).

**Table 3:** Association between retinal vascular abnormalities and risk factors in rural southwestern Harbin, China

		Focal Retinal Vascular Abnormalities								Generalized Narrowing	
		Focal arteriolar narrowing		Arteriovenous nicking		Arteriolar sheathing		Retinopathy			
		%	OR(95% CI)†	%	OR(95% CI)†	%	OR(95% CI)†	%	OR(95% CI)†	%	OR(95% CI)†
Hypertension	*No*	6.2	1	5.9	1	3.4	1	4.8	1	4.3	1
	*YES*	12.6	2.15 (1.77,2.62)**	12.5	2.22 (1.82,2.71)**	6.8	1.95 (1.51,2.54)**	8.8	1.81 (1.44,2.26)**	8.4	1.98 (1.57,2.50)**
Hyperlipidaemia	*No*	8.0	1	8.6	1	4.6	1	6.1	1	4.9	1
	*YES*	13.4	2.60 (2.03,3.33)**	10.2	1.51 (1.17,1.95)**	6.5	2.51 (1.80,3.50)**	8.8	2.16 (1.62,2.88)**	11.1	4.21 (3.15,5.62)**
Diabetes Mellitus	*No*	9.0	1	8.9	1	5.0	1	6.8	1	6.1	1
	*YES*	10.2	1.09 (0.79,1.52)	9.7	1.08 (0.77,1.51)	4.2	0.76 (0.46,1.24)	5.1	0.75 (0.48,1.17)	7.4	1.19 (0.82,1.75)
Stroke History	*No*	9.0	1	8.9	1	4.9	1	6.4	1	6.2	1
	*YES*	13.4	1.56 (0.86,2.82)	12.4	1.43 (0.78,2.64)	6.2	1.04 (0.69,1.56)	18.6	3.27 (1.93,5.54)**	8.2	1.35 (0.65,2.80)
CHD	*No*	8.6	1	8.3	1	4.9	1	6.4	1	6.1	1
	*YES*	14.0	1.72 (1.31,2.25)**	14.8	1.89 (1.45,2.46)**	5.4	1.24 (0.54,2.87)	8.5	1.26 (0.91,1.76)	7.1	1.13 (0.79,1.62)
Smoking Status	*Never*	7.1	1	6.3	1	4.1	1	5.8	1	3.1	1
	Past	11.4	1.77 (1.40,2.23)**	15.2	2.71 (2.17,3.38)**	7.0	1.75 (1.31,2.34)**	10.0	1.60 (1.25,2.06)**	9.4	3.24 (2.43,4.32)**
	*Current*	12.7	2.17 (1.70,2.77)**	9.1	1.56 (1.19,2.05)**	5.1	1.50 (1.05,2.13)*	5.1	0.90 (0.65,1.27)	12.2	5.13 (3.82,6.90)**
Alcohol Drinking Status	*Never*	7.2	1	7.3	1	4.3	1	5.5	1	4.3	1
	Past	16.4	3.30 (2.63,4.14)**	15.1	2.60 (2.06,3.27)**	7.0	2.23 (1.63,3.05)**	9.8	1.77 (1.35,2.32)**	10.3	3.16 (2.39,4.19)**
	*Current*	9.4	1.71 (1.23,2.38)**	9.2	1.51 (1.08,2.10)*	6.2	2.03 (1.35,3.05)**	8.7	1.79 (1.27,2.54)**	12.3	4.09 (2.96,5.65)**

Footnotes: The associations between retinal vascular abnormalities and risk factors were determined using logistic regressions,

*P < 0.05;**P < 0.01.†OR(95% CI) = Odds ratio and 95% confidence interval, adjusted for age, gender and left/right eyes

CHD:Coronary Heart Disease History

After adjusting for age, gender and left/right eyes, FAN and AVN were positively associated with self-reported diagnosis histories of coronary heart diseases (CHD)(8.6% vs 14.0 and 8.3% vs 14.8%), hyperlipidaemia (8.0% vs 13.4 and 8.6% vs 10.2%), and past or current smoking and alcohol consumption habits (all *P* < 0.01). However, the above two types of retinal lesions were not significantly associated with a self-reported diagnosis history of stroke (9.0% vs 13.4 and 8.9% vs 12.4%, *P* > 0.05) (Table 3). After further adjusting for hypertension, hyperlipidaemia, diabetes mellitus, habits of past or current smoking and alcohol the association between FAN and the self-reported diagnosis history of CHD was no longer were statistically significant (OR, 1.27; 95% CI, 0.95, 1.68). Only AVN was strongly associated with the self-reported diagnosis histories of CHD (OR, 1.44; 95% CI, 1.09, 1.89) (Table 4).

**Table 4:** Logistic regression results of the association between retinal vascular abnormalities and cardiovascular and cerebrovascular diseases in rural southwestern Harbin, China

		Focal Retinal Vascular Abnormalities								Generalized Narrowing	
		Focal arteriolar narrowing		Arteriovenous nicking		Arteriolar sheathing		Retinopathy			
		%	OR(95% CI)†	%	OR(95% CI)†	%	OR(95% CI)†	%	OR(95% CI)†	%	OR(95% CI)†
Stroke History	*No*	9.0	1	8.9	1	4.9	1	6.4	1	6.2	1
	*YES*	13.4	1.02 (0.55,1.90)	12.4	0.99 (0.53,1.84)	6.2	0.74 (0.31,1.76)	18.6	2.05 (1.18,3.57)^**^	8.2	0.91 (0.41,1.99)
CHD	*No*	8.6	1	8.3	1	4.9	1	6.4	1	6.1	1
	*YES*	14.0	1.27 (0.95,1.68)	14.8	1.44 (1.09,1.89)^*^	5.4	0.72 (0.47,1.10)	8.5	0.89 (0.63,1.26)	7.1	0.79 (0.54,1.17)

Footnotes:*P < 0.05;**P < 0.01. †OR(95% CI) = Odds ratio and 95% confidence interval, CHD = Coronary Heart Diseases History;

Adjusted for age, gender, left/right eyes, hypertension, hyperlipidaemia, diabetes mellitus, smoking status and alcohol consumption status

Also after controlling for age, gender, and left/right eyes, retinopathy was positively associated with a self-reported diagnosis history of stroke (6.4% vs 18.6%), with hyperlipidaemia (6.1% vs 8.8%) and with habits of past or current smoking and alcohol consumption habits (all *P* < 0.01) (Table 3). After additional adjustment for hypertension, hyperlipidaemia, diabetes mellitus, habits of past or current smoking and alcohol consumption, the association with a history of stroke persisted (OR, 2.05; 95% CI, 1.18, 3.57). Retinopathy was not significantly associated with self-reported diagnosis histories of CHD (OR, 0.89; 95% CI, 0.63, 1.26) (Table 4).

AS and GAN were not associated with the self-reported diagnosis of CHD (4.9% vs 5.4 and 6.1% vs 7.1%, *P* > 0.05) and stroke (4.9% vs 6.2 and 6.2% vs 8.2%, *P* > 0.05) but were independently associated with hyperlipidaemia (4.6% vs 6.5 and 4.9% vs 11.1%), past or current smoking and alcohol consumption habits. For all types of retinal lesions, no significant associations were found between the subjects with or without the presence of diabetes mellitus (P > 0.05).

### Reproducibility of RVAs assessment

Testing the interobserver and intraobserver variability of the assessment, the *k* values were 0.73 to 0.80 for the interassessment and 0.65 to 0.75 for intraobservation of FAN, AVN, AS, retinopathy, and GAN.

## Discussion

Distinguishing from the survey areas of previous studies in Asia (e.g., Beijing [14] and Singapore [24]) and western countries [1–13], the county of Shuangcheng was selected for study to represent the typical environmental region, which was characterized by a cold climate (average 4.5°Cyearly, approach to subfrigid zone), lower elevation (approximately 200 m), farming communities, and plains. This study provides vital epidemiological data on the prevalence of retinal microvascular abnormalities and their associations with CCVds in this environment. Each retinal outcome was determined by the assessment of retinal digital images. First, we found that the overall prevalence of retinal microvascular abnormalities in the present population was relatively higher but the prevalence of retinopathy was lower than those reported in other regions of the world [3, 14, 25–27]. Our finding adds to existing data, details retinal microvascular abnormalities in a rural population in a region of northeast China with a low altitude and cold climate. Second, we showed that retinopathy was associated with a self-reported diagnosis of a stroke, while AVN was associated with a self-reported diagnosis of CHD; but FAN, GAN and AS were not consistently related to the self-reported diagnosis of CHD or previous cerebrovascular events such as stroke. Thus, an examination of retinal microvascular characteristics may offer clues regarding CCVds and could be a potentially novel biomarker of CCVds risk.

In the present study, among the total sample, the prevalence of FAN, AVN, AS, retinopathy, and GAN were 9.1, 8.9, 6.6, and 6.2%, respectively. In comparison, using almost the same assessment methods, the prevalence rates of FAN, AVN, AS and GAN were 6.3, 6.6, 4.8, and 4.3% respectively, in the Beijing Eye Study [14] (Chinese population: 40–101 years of age, including those with diabetes). The prevalence of RVAs in the present study was relatively higher than that of the Beijing Eye Study. The prevalence rates of FAN, AVN, AS and retinopathy in persons without diabetes were 9.0, 8.9, 5.0, and 6.8% respectively in the present study. In comparison, the ARIC study [3], which examined a non-diabetic study population aged 48 to 73 years, found that the prevalence rates were 7.3% for FAN, 6.0% for AVN, and 4.0% for retinopathy. And the Cardiovascular Heart Study (CHS) [28], which also included a non-diabetes population aged 69 to 97 years found that the prevalence rates were 9.6% for FAN, 7.7% for AVN, and 8.3% for retinopathy. Obviously, the prevalence of RVAs in the present study was roughly equivalent to the data of the CHS study but was relatively higher than that of the ARIC study. First, these differences might result from sample selection and population characteristics (e.g., the average age [3, 28, 29] and the frequency of hypertension [29–33] among these studies). Second, the average latitude of the survey area was relatively high (Fig. 1), and the average annual temperature was approximately 4.5 °C, stimulation by cold air is a precipitating factor for CCVds [34, 35]. Presumably, this factor may be related to the high frequency of RVA in this area. In addition, our study employed a 40° non-mydriatic camera to obtain fundus images and used the digital images to grade retinal lesions in the present study. These methods differ from those used in previous studies and thus may have also contributed to the difference between our results and previous findings.

Notably, the prevalence of retinopathy (5.1%) in persons with diabetes was lower than that in Chinese in Beijing (27.9%, ≥45 years) [25], Koreans (15.8%,≥40 years) [26], and Chinese in rural Handan (43.1%, ≥30 years) [27]. The specific reasons for the low prevalence were not clear, in addition to different examination techniques and the grading systems, the prevalence was presumably associated with a still lower living standard and employment involving mainly physical labour. Interestingly, for all types of retinal lesions, no significant differences were detected between the subjects with and without diabetes of the whole sample, which is consistent with the Beijing study. That is to say, although retinopathy (e.g., microaneurysms, haemorrhages, soft exudates or cotton wool spots, hard exudates, etc.) is a landmark of diabetic retinopathy, it is still common in subjects without diabetes who are over 50 years old. We should be cautious in diagnosing diabetic retinopathy, especially in elderly patients.

After stratifying the population by age and gender (Table 2), males and older subjects tended to have more frequent RVAs of all types than their counterparts. These findings were consistent with those of previous reports such as those from the ARIC study [3] and the National Health and Nutrition Examination Survey (NHANES) [36] but different from those of the Beijing Eye study [14] which even showed that AS was more frequent in females than in males.

The focus of the present study was the correlation between RVAs and CCVds, which was discussed respectively. In general, after adjusting for age, gender and left/right eyes, FAN, and AVN were found to be associated with a self-reported diagnosis histories of CHD (OR, 1.72; 95% CI, 1.31, 2.25; and OR, 1.89; 95% CI, 1.45, 2.46, respectively), and its risk factors (e.g., hypertension, hyperlipidaemia, and habits of past/current smoking or alcohol consumption), and when we further adjusted for these above risk factors, the FAN association with CHD disappeared, suggesting that FAN was only related to the risk factors of CHD but was not related to CHD itself. And the AVN-CHD association was attenuated but still existed (OR, from 1.89 to 1.44) (Tables 3, 4), suggesting that the changes in AVN could partly reflect the changes in the cardiac macrovasculature in addition to the microvasculature. In the same way, when adjusted for age, gender and left/right eyes, retinopathy was associated with a self-reported diagnosis history of stroke (OR, 3.27; 95% CI, 1.93,5.54) and its risk factors (hypertension, hyperlipidaemia, habits of past or current smoking, habits of past or current alcohol consumption). When further adjusted for these above risk factors, the retinopathy–stroke association also weakened (OR, from 3.27 to 2.05) but still existed. Thus, these data suggest that a point-to-point association between retinal vascular changes and CCVds may exist (e.g., the AVN-CHD association or retinopathy-stroke association). A multicentre study even demonstrated that different retinopathy signs were associated with specific stroke subtypes [11]; for example, retinal arteriolar narrowing was associated with lacunar stroke, whereas retinal haemorrhages were linked with cerebral haemorrhages [6–8, 11, 36]. However, the results of the CHS study [28] showed that only retinopathy was associated with prevalent CHD and stroke and the results of the Beijing Eye Study [14] showed that RVAs were not related to the self-reported diagnosis of CHD or previous cerebrovascular events such as stroke. These inconsistent results might be due to the different assessment methods and grading thresholds among these studies.

The rest, except for retinopathy, of the RVAs were not related to the self-reported diagnosis history of stroke, but were associated with its risk factors; AS, retinopathy and GAN were not related to the self-reported diagnosis history of CHD, but were associated with the risk factors of CHD (Table 3). Although RVA and CVDs share some of the same risk factors, they are different in structure. Certain types of retinal microvascular abnormalities appear to be associated with systemic processes that are different from those associated with macrovascular changes (e.g., structural, and pathological features), supporting the ARIC [3] and CHS study [28] findings in middle-aged people.

Some factors may have influenced our evaluation of the prevalence of RVAs and their possible correlation with CCVds. First, images were not obtained from some of the participants, and some photos could not be evaluated due to refractive interstitial opacity. A relatively high proportion of these images were obtained from elderly patients, who generally have more RVAs. Second, the evaluation of retinal abnormalities was performed manually, which may lead to the relatively low *κ* values, for intraobserver and interobserver variation. Third, we used questionnaires to collect the histories of diseases as the basis of the prevalence estimates of CCVds. This may underestimate the associations with systemic diseases. Fourth, for the assessment of GAN, considering the engorged and tortuous of veins may caused by other reasons, such as branch or central retinal vein occlusion, so these eyes were not defined as GAN. This would lead to an underestimation of the prevalence of GAN. Last, due to the use of a cross-sectional study design rather than a cohort study design, the current study could not elucidate the evolution of RVAs and their real-time relationship with systemic diseases.

What is the innovative points and clinical significance of the present study? Previous studies mostly tended to describe the association between RVAs and CCVds in general or to explore the association between retinal vessel diameter and CCVds with image analysis technology [5–11, 36–39]. In this study, we found that point-to-point associations might exist between specific types of RVAs and CCVds(e.g., the AVN-CHD association or retinopathy-stroke association). If such associations could be demonstrated as stable, we may need to pay more attention to the correlation between specific types of RVAs and CCVds. Thus, the prediction of CCVds may become more targeted, especially in rural primary hospitals where medical equipment was relatively scarce. In addition, a semi-quantitative classification method for RVAs was used in this study. Due to the use of digital fundus photographs and standard evaluation system (ARIC), the variability among observers is significantly lower than that of the use of direct fundoscopic examination [40]. Moreover, because this method is low-cost and efficient and it does not need professional technicians and expensive professional software, it could be popularized and implemented in primary hospitals in undeveloped regions. However, due to the relatively limited geographical scope of this study, future clinical studies involving large samples and multiple regions are needed clarify the stability of this correlation between specific types of RVAs and CCVds.

## Conclusion

This study details retinal microvascular abnormalities in a rural population in a region of northeast China with a low altitude and cold climate. The overall prevalence of retinal microvascular abnormalities in this population was relatively higher than that reported in other regions of the world. However, the prevalence of retinopathy was low. Retinopathy was associated with a self-reported diagnosis of stroke history and AVN was associated with the self-reported diagnosis of CHD; FAN, GAN and AS were not consistently associated with the diagnoses of CHD or previous cerebrovascular events such as stroke. Thus, an examination of retinal microvascular characteristics may offer clues to CCVds and could be potentially be considered as of a novel biomarker of CCVd risk.

## Data Availability

The datasets used and/or analysed during the current study are available from the corresponding author on reasonable request.

## Abbreviations

RVAs: Retinal vascular abnormalities
GAN: Generalized arteriolar narrowing
FAN: Focal arteriolar narrowing
AVN: Arteriovenous nicking
AS: Arteriolar sheathing
CCVds: Cardiovascular and Cerebrovascsular diseases
ARIC: Atherosclerosis Risk in Communities study
CHS: The Cardiovascular Health Study
DM: Diabetes mellitus
HP: Hypertension
OR: Odds Ratio

## References

[1] Yang SH, Dou KF, Song WJ (2010). Prevalence of diabetes among men and women in China. N Engl J Med.

[2] Jeganathan VS, Sabanayagam C, Tai ES (2009). Effect of blood pressure on the retinal vasculature in a multi-ethnic Asian population. Hypertens Res.

[3] Hubbard LD, Brothers RJ, King WN, Clegg LX, Klein R, Cooper LS, Sharrett AR, Davis MD, Cai J (1999). Methods for evaluation of retinal microvascular abnormalities associated with hypertension/sclerosis in the atherosclerosis risk in communities Study. Ophthalmology.

[4] Wong TY, Klein R, Klein BE (2001). Retinal microvascular abnormalities and their relationship with hypertension, cardiovascular disease, and mortality. Surv Ophthalmol.

[5] Wong TY, Kamineni A, Klein R, Sharrett AR, Klein BE, Siscovick DS (2006). Quantitative retinal venular caliber and risk of cardiovascular disease in older persons: the cardiovascular health Study. Arch Intern Med.

[6] Yatsuya H, Folsom AR, Wong TY, Klein R, Klein BE, Sharrett AR; ARIC Study Investigators. Retinal microvascular abnormalities and risk of lacunar stroke: atherosclerosis risk in communities Study. Stroke 2010;41:1349–1355. doi: 10.1161/STROKEAHA.110.580837. 10.1161/STROKEAHA.110.580837 PMC2894269 20522816

[7] Baker ML, Hand PJ, Wong TY, Liew G, Rochtchina E, Mitchell P, et al*.* Multi-Centre retinal stroke Study group. Retinopathy and lobar intracerebral hemorrhage: insights into pathogenesis. Arch Neurol 2010;67:1224–1230. doi: 10.1001/archneurol.2010.249. 10.1001/archneurol.2010.249 20937950

[8] Baker ML, Hand PJ, Liew G, Wong TY, Rochtchina E, Mitchell P, et al*.* Multi-Centre retinal stroke Study group. Retinal microvascular signs may provide clues to the underlying vasculopathy in patients with deep intracerebral hemorrhage. Stroke 2010;41:618–623. doi: 10.1161/STROKEAHA.109.569764. 10.1161/STROKEAHA.109.569764 20167920

[9] Wong TY, Klein R, Couper DJ, Cooper LS, Shahar E, Hubbard LD, et al*.* Retinal microvascular abnormalities and incident stroke: the atherosclerosis risk in communities Study. Lancet 2001;358:1134–1140. doi: 10.1016/S0140-6736(01)06253-5. 10.1016/S0140-6736(01)06253-5 11597667

[10] Mitchell P, Wang JJ, Wong TY, Smith W, Klein R, Leeder SR. Retinal microvascular signs and risk of stroke and stroke mortality. Neurology 2005;65:1005–1009. doi: 10.1212/01.wnl.0000179177.15900.ca. 10.1212/01.wnl.0000179177.15900.ca 16217050

[11] Lindley RI, Wang JJ, Wong MC, Mitchell P, Liew G, Hand P (2009). Multi-Centre retina and stroke Study (MCRS) collaborative group. Retinal microvasculature in acute lacunar stroke: a cross-sectional study. Lancet Neurol.

[12] Wong TY, Klein R, Sharrett AR, Duncan BB, Couper DJ, Tielsch JM (2002). Retinal arteriolar narrowing and risk of coronary heart disease in men and women: the atherosclerosis risk in communities Study. JAMA.

[13] Duncan BB, Wong TY, Tyroler HA, Davis CE, Fuchs FD (2002). Hypertensive retinopathy and incident coronary heart disease in high risk men. Br J Ophthalmol.

[14] Wang S, Xu L, Jonas JB (2006). Retinal vascular abnormalities in adult Chinese in rural and urban Beijing: the Beijing eye Study. Ophthalmology.

[15] Wong TY, Knudtson MD, Klein R, Klein BE, Meuer SM, Hubbard LD. Computer-assisted measurement of retinal vessel diameters in the beaver dam eye Study: methodology, correlation between eyes, and effect of refractive errors. Ophthalmology 2004;111(6): 1183–1190. doi: 10.1016/j.ophtha.2003.09.039. 10.1016/j.ophtha.2003.09.039 15177969

[16] Cheung CY, Hsu W, Lee ML, et al. A new method to measure peripheral retinal vascular caliber over an extended area. Microcirculation 2010;17(7):495–503. doi: 10.1111/j.1549-8719.2010.00048.x. 10.1111/j.1549-8719.2010.00048.x 21040115

[17] Sun C, Wang JJ, Mackey DA, Wong TY. Retinal vascular caliber: systemic, environmental, and genetic associations. Surv Ophthalmol 2009;54(1):74–95.doi: 10.1016/j.survophthal.2008.10.003. 10.1016/j.survophthal.2008.10.003 19171211

[18] Li S, Xu J, He M, et al. A survey of blindness and cataract surgery in Doumen County, China. Ophthalmology 1999;106:1602–1608. doi: 10.1016/S0161-6420(99)90459-1. 10.1016/S0161-6420(99)90459-1 10442910

[19] Hu C, Zhao JL, Liu XL (1988). The epidemiological study of the blindness and low vision in Shunyi County of Beijing. Chin J Ophthalmol.

[20] Zhang Y, Wang H, Liu P, et al. Prevalence of blindness and low vision: a study in the rural Heilongjiang Province of China. Clin Exp Ophthalmol 2012; 40(5):484–489. doi:10.1111/j.1442-9071.2011.02682.x. 10.1111/j.1442-9071.2011.02682.x 21902783

[21] The major data communique of the sixth national census, National Bureau of Statistics of the People's Republic of China. http://www.stats.gov.cn/tjsj/pcsj/.

[22] Liang YB, Friedman DS, Wong TY, Wang FH, Duan XR, Yang XH, et al. (2009) Handan eye Study group. Rationale, design, methodology, and baseline data of a population-based study in rural China: the Handan eye Study. Ophthalmic Epidemiol 16:115–127. doi: 10.1080/09286580902738159. 10.1080/09286580902738159 19353400

[23] Expert Committee on the Diagnosis and Classification of Diabetes Mellitus (1997). Report of the Expert Committee on the Diagnosis and Classification of Diabetes Mellitus. Diab Care.

[24] Cheung CY, Tay WT, Ikram MK, Ong YT, De Silva DA, Chow KY, et al. Retinal Microvascular Changes and Risk of Stroke: The Singapore Malay Eye Study. Stroke. 2013; 9;44(9):2402–2408. doi: 10.1161/STROKEAHA.113.001738. 10.1161/STROKEAHA.113.001738 23868266

[25] Jee D, Lee WK, Kang S. Prevalence and risk factors for diabetic retinopathy: the Korea National Health and nutrition examination survey 2008–2011. Invest Ophthalmol Vis Sci 2013; 54:6827–6833. doi:10.1167/iovs.13-12654. 10.1167/iovs.13-12654 24065813

[26] Wang FH, Liang YB, Zhang F, et al. Prevalence of diabetic retinopathy in rural China: the Handan eye Study. Ophthalmology 2009; 116: 461–467. doi:10.1016/j.ophtha.2008.10.003. 10.1016/j.ophtha.2008.10.003 19168222

[27] Wong TY, Klein R, Sharrett AR, et al. Retinal microvascular abnormalities and cognitive impairment in middle-aged persons: the atherosclerosis risk in communities Study. Stroke 2002; 33:1487–1492. doi:10.1161/01.str.0000016789.56668.43. 10.1161/01.str.0000016789.56668.43 12052979

[28] Wong TY, Klein R, Sharrett AR, et al. The prevalence and risk factors of retinal microvascular abnormalities in older people: the cardiovascular health Study. Ophthalmology 2003; 110: 658–666. doi: 10.1016/S0161-6420(02)01931-0. 10.1016/S0161-6420(02)01931-0 12689883

[29] Klein R, Klein BE, Moss SE, Wang Q (1994). Hypertension and retinopathy, arteriolar narrowing, and arteriovenous nicking in a population. Arch Ophthalmol.

[30] Cheung CY, Tay WT, Ikram MK, Ong YT, De Silva DA, Chow KY, et al*.* Retinal microvascular changes and risk of stroke: the Singapore Malay eye Study. Stroke 2013;44(9):2402–2408. doi:10.1161/STROKEAHA.113.001738. 10.1161/STROKEAHA.113.001738 23868266

[31] Rizzoni D, Muiesan ML. Retinal vascular caliber and the development of hypertension: a meta-analysis of individual participant data. J Hypertens 2014;32(2):225–227. doi:10.1097/HJH.0000000000000009. 10.1097/HJH.0000000000000009 24430117

[32] Popovic, N ; Radunovic, M ; Badnjar, J ; Popovic, T; Fractal dimension and lacunarity analysis of retinal microvascular morphology in hypertension and diabetes. Microvasc Res 2018; 118, 36–43. doi:10.1016/j.mvr.2018.02.006. 10.1016/j.mvr.2018.02.006 29476757

[33] MartiSoler H, Gonseth S, Gubelmann C, et al. Seasonal variation of overall and mortality: a study in 19 countries from different geographic locations. PLoS One 2014; 9(11):e113500. doi:10.1371/journal.pone.0113500. 10.1371/journal.pone.0113500 PMC4242652 25419711

[34] Jongsik Ha, Joungho Yoon, Ho Kim. Relationship between winter temperature and mortality in Seoul, South Korea, from 1994 to 2006. Sci Total Environ 2009; 407(7):2158–2164. doi:10.1016/j.scitotenv.2008.12.029. 10.1016/j.scitotenv.2008.12.029 19157521

[35] Xie XW, Xu L, Wang YX, Jonas JB (2008). Prevalence and associated factors of diabetic retinopathy. The Beijing eye Study 2006. Graefes Arch Clin Exp Ophthalmol.

[36] Wong TY, Klein R, Sharrett AR (2002). Cerebral white matter lesions, retinopathy, and incident of clinical stroke: the atherosclerosis risk in communities Study. JAMA.

[37] van Hecke MV, Dekker JM, Nijpels G, Moll AC, Heine RJ, Bouter LM, et al*.* Inflammation and endothelial dysfunction are associated with retinopathy: the Hoorn Study. Diabetologia 2005;48:1300–1306. doi:10.1007/s00125-005-1799-y. 10.1007/s00125-005-1799-y 15918015

[38] Wong TY, Klein R, Sharrett AR (2002). Retinal arteriolar narrowing and risk of diabetes in middle-aged persons. JAMA.

[39] Anyfanti P, Triantafyllou A, Gkaliagkousi E, Koletsos N, Athanasopoulos G, Zabulis X, et al*.* Retinal vessel morphology in rheumatoid arthritis: Association with systemic inflammation, subclinical atherosclerosis, and cardiovascular risk. Microcirculation 2017;24(8). doi:10.1111/micc.12417. 10.1111/micc.12417 28926162

[40] Ikram MK, Borger PH, Assink JJ, Jonas JB, Hofman A, De Jong PT (2002). Comparing ophthalmoscopy, slide viewing, and semiautomated systems in optic disc morphometry. Ophthalmology..

